# No up-regulation of the phosphatidylethanolamine N-methyltransferase pathway and choline production by sex hormones in cats

**DOI:** 10.1186/s12917-015-0591-6

**Published:** 2015-11-09

**Authors:** Chiara Valtolina, Arie B. Vaandrager, Robert P. Favier, Joris H. Robben, Maidina Tuohetahuntila, Anne Kummeling, Isabelle Jeusette, Jan Rothuizen

**Affiliations:** Department of Clinical Sciences of Companion Animals, Faculty of Veterinary Medicine, Utrecht University, Yalelaan 108, 3584 CM Utrecht, The Netherlands; Department of Biochemistry and Cell Biology, Faculty of Veterinary Medicine and Institute of Biomembranes, Utrecht University, Yalelaan 2, 3584 CM Utrecht, The Netherlands; Research and Development, Affinity Petcare, Pl. Xavier Cugat, 2 Edificio D, 3ª, Planta, 08174 St. Cugat del Vallès, Barcelona, Spain

**Keywords:** Cats, Hepatic lipidosis, Choline, PEMT, Oestrogen

## Abstract

**Background:**

Feline hepatic lipidosis (FHL) is a common cholestatic disease affecting cats of any breed, age and sex. Both choline deficiency and low hepatic phosphatidylethanolamine N-methyltransferase (PEMT) activity are associated with hepatic lipidosis (HL) in humans, mice and rats. The PEMT expression is known to be upregulated by oestrogens, protecting the females in these species from the development of HL when exposed to choline deficient diets. The aim of the present study was to evaluate the influence of sex hormones on choline synthesis via the PEMT pathway in healthy male and female cats before and after spaying/neutering, when fed a diet with recommended dietary choline content.

**Results:**

From six female and six male cats PEMT activity was assayed directly in liver biopsies taken before and after spaying/neutering, and assessed indirectly by analyses of PEMT–specific hepatic phosphatidylcholine (PC) species and plasma choline levels. Hepatic PEMT activity did not differ between intact female and male cats and no changes upon spaying/neutering were observed. Likewise, no significant differences in liver PC content and PEMT-specific polyunsaturated PC species were found between the sexes and before or after spaying/neutering.

**Conclusion:**

These results suggest that choline synthesis in cats differs from what is observed in humans, mice and rats. The lack of evident influence of sex hormones on the PEMT pathway makes it unlikely that spaying/neutering predisposes cats for HL by causing PC deficiency as suggested in other species.

## Background

Feline hepatic lipidosis (FHL) is a common cholestatic disease affecting cats. It is considered the consequence of prolonged anorexia and subsequent dramatic lipolysis [1–3]. Feline hepatic lipidosis is characterized by an excessive accumulation of triglycerides (TAG) in hepatocytes. Although the exact mechanisms remain elusive, there is clearly an imbalance between the influx of fatty acids (FA) derived from peripheral fat stores and *de novo* synthesis of FA in the liver on the one hand, and the rate of hepatic FA oxidation and the efflux of hepatic TAGs via very-low density lipoprotein (VLDL) on the other hand [1–3]. In cats with FHL, serum TAG levels significantly increase with the greatest distribution into the VLDL fraction. It has been suggested that a lower capacity to excrete VLDL plays a role in the development of FHL [4].

Secretion of VLDL is dependent on the rate of hepatic phosphatidylcholine (PC) synthesis. Phosphatidylcholine is synthetized in the liver by two pathways, the Kennedy or cytidine diphosphate-choline (CDP-choline) pathway and the phosphatidylethanolamine N-methyltransferase (PEMT) pathway. The Kennedy pathway produces more saturated and mono-unsaturated acyl chain-containing PC, whereas the PEMT pathway synthesizes PC with a long highly unsaturated acyl chain (HUFA) (PC 38:6 and PC 40:6) [5]. The Kennedy pathway is the major route for PC synthesis and is present in all mammalian tissues. The synthesis of PC via this pathway depends on the intake of choline [6]. The PEMT pathway is independent of choline intake and converts phosphatidylethanolamine (PE) to PC via the transfer of three methyl-groups from S-adenosylmethionine [7, 8]. The PEMT pathway is liver-specific and is estimated to account for approximately 20–40 % of the newly synthesized PC in the liver in humans, mice and rats [9–11]. In premenopausal women, intact female mice and rats it has been estimated that a larger fraction of PC is made in the liver via the methylation of PE, than in men, postmenopausal women and sterilised mice and rats [9–11].

The role of choline in hepatic lipidosis (HL) has been evaluated in humans [12], cats [13–16] and mice [17]. Both choline deficiency and low PEMT activity have been associated with HL [18–22]. Suboptimal concentrations of dietary choline are associated in the cat with a diminished capacity of the liver to synthesize PC resulting in accumulation of lipids in the liver [13–15].

In humans, mice and rats PEMT expression and activity is known to be upregulated by oestrogens. This explains why premenopausal and intact females are less choline dependent than males or postmenopausal/spayed females [9, 11, 23–25]. Pre-menopausal women have a 30–50 % higher capacity than men to form PC *de novo* via the PEMT pathway [23, 24]. Premenopausal women and female rats on a choline deficient diet are less likely to develop HL than their male counterparts. Postmenopausal women and castrated female mice with less PEMT activity are considered more susceptible to develop PC deficiency and, subsequently, HL [11, 24].

Center et al. have reported that female cats are more affected than male cats [3], but it is more commonly reported that FHL has no breed, age or sex predisposition [1, 2]. An explanation for an occasionally proposed sex predisposition in FHL has never been convincingly described.

As demonstrated in other species, the loss of oestrogen influence could cause spayed female cats to have a lower capacity for PC synthesis than intact females. If so, spaying female cats, a practice widely performed in all western countries, could be a predisposing factor for female cats to develop FHL.

The aim of this study was to analyse the lipid metabolism in healthy cats before and after spaying/neutering and subsequently the influence of sex hormones on PC synthesis via the PEMT pathway.

## Results

### Cats

Six intact females with a median age of 20.5 months (range: 6.0–84.0) and 6 intact males with a median age of 7.0 months (range: 6.0–9.0) were enrolled. Median body weight of the female cats was 3.0 kg (range: 2.6–3.9); median body weight of the male cats was 4.0 kg (range: 3.5–4.5).

All cats accepted the diet without any problems. Results of the complete blood count (CBC), biochemistry and coagulation profile of all twelve cats, analysed following standard chemical analyses, were within reference intervals (results not shown). The surgical procedures and ultrasound guided liver biopsies were without incident in all cats. The cats recovered uneventfully from anaesthesia and were discharged the day after.

### Histological analysis

Histological evaluation of the liver biopsies performed in each subject (before and after spaying/neutering) at 4 weeks and at 8 weeks from the start of the diet revealed no histological changes compatible with hepatic lipidosis or other histological changes.

### Hormone analysis

The results of the sex hormones measurements in the blood of the male and female cats pre and post spaying/neutering are reported in Table 1. Oestrogen levels dropped significantly after spaying (*P* = 0.041), indicating successful spay in all female cats. In one female cat, both before and after spaying, the oestrogen levels were below the detection limit of the test.

**Table 1:** Plasma oestrogen and testosterone levels (mean ± SD) before and after spaying/neutering

	Group 1 (male intact ) (*n* = 6)	Group 2 (male neutered) (*n* = 6)	Group 3 (female intact) (*n* = 6)	Group 4 (female spayed) (*n* = 6)
Testosterone^a^ (pmol/L)	824.3 ± 929.4	36.6 ± 17.0		
Oestrogen^b^ (pmol/L)			9.6 ± 6.4	3.0 ± 1.0

^a^ *P* < 0.001 male intact versus neutered

^b^ *P* < 0.041 female intact versus spayed

The testosterone level dropped significantly (*P* = 0.001) in male cats, indicating successful neutering.

### Plasma choline and liver PEMT activity analysis

Free plasma choline levels were not statistically different between intact female cats compared to intact males (*P* = 0.76) (Table 2). After spaying the choline levels increased in female cats by 35 % to levels similar to those in male cats pre and post neutering (Table 2), but the increase was not significant.

**Table 2:** Plasma choline levels and hepatic indicators of phosphatidylcholine (PC) metabolism (mean ± SD) in male and female cats before and after spaying/neutering

	Group 1 (male intact) (*n* = 6)	Group 2 (male neutered) (*n* = 6)	Group 3 (female intact) (*n* = 6)	Group 4 (female spayed) (*n* = 6)
plasma choline (μM)	1.9 ± 0.8	1.8 ± 0.7	1.5 ± 0.3	2.0 ± 0.5
liver PEMT^a^ activity (nmol/mg protein/h)	0.24 ± 0.18	0.19 ± 0.11	0.15 ± 0.09	0.21 ± 0.16
liver PC content (nmol/mg protein)	24.2 ± 3.8	24.1 ± 3.2	27.5 ± 3.1	24.1 ± 2.3
liver PC 38:6^b^ (% of total PC)	3.3 ± 1.1	3.7 ± 1.6	4.2 ± 0.4	3.8 ± 0.5
liver PC 40:6^b^ (% of total PC)	3.3 ± 1.3	4.2 ± 2.3	4.1 ± 0.6	3.7 ± 0.6
liver PC/PE^c^ ratio	3.4 ± 0.4	3.1 ± 0.3	4.0 ± 1.4	4.0 ± 1.5

^a^ *PEMT* phosphatidylethanolamine N-methyltransferase

^b^PC species implicated in PEMT activity i.e. PC containing long highly unsaturated acyl chains

^c^ *PE* phosphatidylethanolamine

Activity of PEMT varied considerably between samples and did not differ significantly between male and female cats before or after spaying/neutering, and no effects of spaying/neutering were found in either group (Table 2). No correlation between the individual plasma choline levels and PEMT activity was observed (results not shown).

As presented in Table 2, no significant differences were observed in total liver PC and the PEMT-specific PC species *i.e.* long chain highly unsaturated (HUFA) PC species, PC 38:6 (PC 16:0, 22:6) and PC 40:6 (PC 18:0, 22:6), and the PC/PE ratio between male and female cats before or after spaying/neutering.

## Discussion

Choline deficiency and low hepatic PEMT activity have been associated with HL in humans, mice and rats. Pre-menopausal and intact females in these species appear better protected from the development of lipidosis when exposed to a choline deficient diet. The up-regulation by oestrogens increases their capacity to synthesise PC via the PEMT pathway, reducing the dependency on choline intake [11, 24, 25]. Also in cats, the importance of choline in the development of HL has been substantiated in supplementation and deficiency studies [13–16]. This study could not demonstrate an effect of a reduced exposure to oestrogens on the PEMT pathway and the level of PEMT-specific PC species in cats. Therefore, this study does not support the hypothesis that a down-regulated PEMT pathway, as result of spaying/neutering, is a predisposing factor for the development of HL when cats are fed at recommended choline levels [26].

The choline levels in the plasma (range 1–3.2 μM) were low compared to what has been established in humans (range 7 – 20 μM) [12] and the earlier reported choline levels in cat plasma (3–7 μM) [27]. However, the latter range has been established with a different assay method [12, 27]. The lower choline levels in the plasma may have been influenced by assay interference of substances in cat plasma.

The choline levels in female intact cats tended to be lower compared to the levels in spayed female cats and male cats. Nevertheless, this finding should be interpreted with caution based on the overall low choline levels close to the lower limit of quantitation. We have no clear explanation for the rise in plasma free choline levels after spaying in female cats, but it may suggest an effect of oestrogen on either choline uptake from the diet or on the conversion of choline to other metabolites like acetylcholine, betaine or phosphatidylcholine.

In contrast to what has been reported in humans, mice and rats, the PEMT activity tended to be lower in intact female cats than in male cats and spayed female cats. Unexpectedly, there was no higher PEMT activity in intact females before spaying, nor a decrease in PEMT related parameters after spaying. This is in contradiction to what is anticipated when oestrogens would up-regulate the PEMT pathway. The low PEMT activity in intact female cats may not have reached significance because of the large variation in activity between samples. This may be partly due to the relatively low signals in the respective assays, making them more susceptible to variation in background noise.

To further elucidate the activity of the PEMT pathway, PC 38:6 and PC 40:6 and the PC/PE ratio were evaluated. Phosphatidylcholine 38:6 and PC 40:6 are products of the PEMT pathway and therefore reflect indirectly the activity of this pathway [5, 9]. The PC/PE ratio is also considered a proxy for the PEMT pathway although it may also be influenced by the rate of the PC and PE synthesis and breakdown by other pathways [8]. It is suggested that the higher the PC/PE ratio, the higher the PEMT activity should be, as more PE would be converted to PC in the hepatocytes via three sequential steps of methylation [5, 7, 9]. Also, in these indirect parameters, there were no significant differences between the groups that could suggest an effect of oestrogen levels on activity of the PEMT pathway.

As the study was performed in client-owned cats, a diet with recommended choline content and not a choline restricted diet was used. With sufficient choline in the diet to allow PC to be formed via the Kennedy pathway, the expression and activity of the PEMT pathway may not have been fully stimulated. However, as it has been demonstrated in humans, mice and rats, the PEMT pathway is also active with diets that contain sufficient choline for the specific species, contributing to 20–40 % of the PC produced [9–11].

This study was performed in a relatively small series of animals. A power analysis indicated that 6 cats per group would have been sufficient to evaluate the hormonal effect on the PEMT pathway. The power analysis was based on the assumption that changes needed to be large to be considered clinically relevant. Nevertheless, it cannot be excluded that significant differences would be found with larger numbers.

It is possible that the difference of prevalence of lipidosis in female cats compared to male cats might be associated to a specific female lipid profile [3]. Further evaluation of lipid profiling of liver and plasma of healthy female and male cats before and after spaying/neutering may help to clarify potential sex difference in cats.

## Conclusion

Phosphatidylcholine synthesis in cats, when fed a recommended amount of choline in the diet, seems to differ from what has been observed in humans, mice and rats, with no evidence of influence of sex hormones on the PEMT pathway. Current recommendations for diet choline levels are adequate for spayed female cats and it is unlikely that castration predisposes cats for HL by causing PC deficiency as suggested in other species when recommended dietary choline level are provided.

## Methods

This study was approved by the Committee for the Ethical Care of Animals of the Utrecht University.

### Cats and study design

Client-owned cats (six intact females, six intact males) admitted to the Department of Clinical Science of Companion Animals of the Faculty of Veterinary Medicine, Utrecht University (DSCA) for spaying/neutering were eligible to enter the study. Informed owner written consent was obtained prior to enrolment of all cats. The cats had to be in good clinical health during the study based on general physical examination performed by one of the authors (CV; veterinary specialist (Diplomate of the American and European College of Veterinary Emergency and Critical Care)), a CBC, and biochemistry test results. Coagulation parameters needed to be within reference range in preparation for the liver biopsy. The health status was checked twice: on admission for the castration and 4 weeks later prior to the second liver biopsy.

Cats enrolled for the study received the same study diet for 8 weeks. Four weeks after the start of the diet, blood samples and liver biopsies were taken. Following confirmation of good health status and sampling, the cats were neutered or spayed. Four weeks after the surgical procedure (8 weeks after the start of the diet) blood and liver tissue sampling were repeated.

For the results analysis and interpretation, four groups were considered: intact males (before neutering, *n* = 6), intact females (before spaying, *n* = 6), spayed female (after spaying, *n* = 6), neutered male (after neutering, *n* = 6).

### Diet

During the study, all the cats were fed a commercially available complete dry diet for adult cat (Affinity Petcare, Barcelona, Spain), provided in 500 g bags and as snacks (Table 3). Although originally formulated for adult cats, this diet covered recommended nutrients allowance for growth with a margin of 10 % (NRC [26]). The dry diet contained a fixed amount of choline (2400 ppm as fed, 0.65 g/1000 kcal), close to the adequate intake of choline (0.64 g/1000 kcal) as recommended by NRC [26] (Table 3). Owners were given a table with the recommended amount of food to administer to their cat based on its weight (100 kcal/kg body weight^0.67^), either 100 % delivered with the diet or 95 % delivered with the diet and 5 % with snacks with similar food composition (Table 3). The energy allowance recommended was the one as published by NRC [26] for adult cats [26]. Compared to NRC [26] recommendations for entire growing kittens between 6 and 12 months, it could have been 10 to 13 % lower (Table 3). Owners were instructed to report any lack of food intake or palatability to one of the authors (CV).

**Table 3:** Nutrient composition of the complete dry diet and snacks as used in this study and the National research Council (NRC) requirements for adult cats and kittens

Nutrients	Analysis method		Diet	Snack	Diet	Snack		Diet	Snack	Adult cat	Adult cat	800 g kitten
		Unit	(as fed)	(as fed)	(DM^a^)	(DM)	Unit					
Moisture	CE N°152/2009	g/100 g	7.2	8.1								
Protein	AOAC 990.03/NF V18-120	g/100 g	28.7	28.2	30.9	30.7	g/1000 kcal	77.8	74.5	40.0	50.0	56.3
Fat	AOAC 954.02	g/100 g	11.8	12.7	12.7	13.8	g/1000 kcal	32.1	33.5	ND^b^	22.5	22.5
Ash	AOAC 942.05	g/100 g	7.6	5.8	8.1	6.3	g/1000 kcal	20.5	15.3			
Crude fiber	AOCS Ba6-05	g/100 g	2.7	2.0	2.9	2.2	g/1000 kcal	7.3	5.3			
Nitrogen free extract	calculated	g/100 g	42.1	43.2	45.3	47.0	g/1000 kcal	114.0	114.2			
Starch	DIR. 72/199/CEE	g/100 g	30.5	ND	32.9	ND	g/1000 kcal	82.7	ND			
Methionine	CE N° 152/2009	g/100 g	0.45	0.47	0.48	0.51	g/1000 kcal	1.22	1.24	0.34	0.43	1.10
Cystine	CE N° 152/2009	g/100 g	0.64	0.65	0.69	0.71	g/1000 kcal	1.73	1.72			
methionine + cystine	CE N° 152/2009	g/100 g	1.09	1.12	1.17	1.22	g/1000 kcal	2.95	2.96	0.68	0.85	2.20
Choline	Ionic Cromatography	g/100 g	0.24	0.24	0.26	0.26	g/1000 kcal	0.65	0.63	0.51	0.64	0.64
Taurine	HPLC	g/100 g	0.07	0.07	0.08	0.08	g/1000 kcal	0.20	0.18	0.08	0.10	0.10
Betaine			ND	ND	ND	ND		ND	ND			
Folate (Vitamine B9)	HPLC	mg/kg	2.81	1.93	3.03	2.10	mg/1000 kcal	0.76	0.51	0.15	0.19	0.19
Cobalamine (Vitamine B12)	HPLC	μg/kg	40.00	30.00	43.09	32.64	μg/1000 kcal	10.84	7.93	ND	5.60	5.60
added Pyridoxine (Vitamine B6)	AOCS Ba6-05	mg/kg	10.39	8.55	11.19	9.30	mg/1000 kcal	2.82	2.26	0.50	0.63	0.63
calculated ME (NRC 2006) [26]	calculated	kcal/kg	3689.0	3784.0	3973.9	4117.5						

^a^ *DM* dry matter

^b^ *ND* not determined

### Blood collection

To determine the health status of the cats, blood (2 mL) was collected by jugular venepuncture and divided into three tubes: 0.5 mL in EDTA (CBC), 0.5 mL in heparin (biochemical analysis), 1 mL in citrate (coagulation).

For hormone and choline analysis, blood (4 mL) was collected by jugular venepuncture and divided into two tubes: 2 mL in heparin (hormone), and 2 mL in heparin (choline). The tubes were centrifuged and the plasma was separated. The plasma for choline analysis was immediately stored at −70 °C in cryogenic vials (Corning Inc., NY, USA).

### Hormone analysis

Plasma oestradiol-17β concentration was measured by radioimmunoassay (RIA) (Coat-A-Count TKE; Diagnostic Products Corporation, Los Angeles, CA, USA) according to the manufacturer’s instructions with modifications as described previously [28] and validated for the dog [29]. The intra-assay and interassay coefficients of variability (CV) were 14.0 and 11.8 %, respectively. The lower limit of quantitation was 7 pmol/L.

Plasma testosterone concentration was measured by RIA (Coat-A-Count Total Testosterone; Diagnostic Product Corporation, Los Angeles, CA, USA) according to the manufacturer’s protocol with previously described modifications to increase the sensitivity [30]. The intra-assay and interassay CV were 5 and 6 %, respectively. The lower limit of quantitation was 51 pmol/L.

### Plasma choline analysis

Free choline concentration was determined with a choline detection kit (Biovision, Milpitas, CA, USA) by measuring the absorbance at 570 nm in a 96 well plate according to the manufacturer’s instructions. Due to the low free choline concentrations 100 μL instead of 50 μL of cat plasma was used. The choline signal had to be corrected for a linear increase in non-specific signal observed after the endpoint of the assay. The lower limit of quantitation was 1 μM.

### Anaesthesia and surgical procedure

Premedication consisted of glycopyrrolate (0.01 mg/kg, intramuscularly (IM)), 30 min later followed by midazolam (0.2 mg/kg IM) and ketamine 5 mg/kg IM. Anaesthesia was induced with alfaxalone (1–2 mg/kg intravenously (IV)) after which the cat was intubated. Anaesthesia was maintained with alfaxalone (4–5 mg/kg/h IV) and inhalation of a mixture of air, oxygen and isoflurane. Castration was performed following a routine procedure. Each animal remained hospitalised until the morning after surgery when it was discharged.

### Liver biopsies

Under general anaesthesia, an ultrasound of the liver was performed by a veterinary specialist (Diplomate of the European College of Veterinary Diagnostic Imaging or resident in the same specialty). If no abnormalities were detected in liver structure and echogenicity, two ultrasound guided liver biopsies were taken with a 16 G needle (Super Core Semi-automatic Biopsy Instrument; Angitech, Vancouver, BC, Canada).

One sample was fixated in 4 % formalin and another was rinsed in normal saline (NaCl 0.9 %), rapidly frozen in liquid nitrogen and stored at −80 °C. Ultrasound of the abdomen was repeated 1–3 h after liver biopsy to evaluate the presence of free abdominal fluid (blood) around the biopsy site.

### Histology analysis

Samples fixed in 4 % neutral-buffered formalin were embedded in paraffin. Sections (3 μm) were cut for routine staining with haematoxylin and eosin (H&E) staining. The slides were reviewed by a specialist in veterinary pathology.

### Liver PEMT activity analysis

For the PEMT activity analyses the frozen liver biopsies were thawed, re-suspended in 350 μL of buffer A (250 mM sucrose, 0.2 mM EDTA, 5 mM DTT, and 10 mM Tris/HCl pH 8.0), and homogenized by mechanical disruption with a pestle tightly fitting to an Eppendorf tube followed by sonication (10 s, amplitude 10 μm). PEMT activity was determined as previously described [31]. Protein was determined in the homogenates by the BCA method Pierce® BCA protein assay kit (Thermo scientific, Rockford, IL, USA) with BSA as standard.

To 100 μL of homogenized liver biopsy, 100 pmol of di-linolenoyl PC (PC (18:3, 18:3)) was added as internal standard. Subsequently, lipids were extracted and separated in a neutral and phospholipid fraction by fractionation on a silica-G column as described [32].

The PEMT activity was also assessed indirectly by analyses of PEMT-specific PC species, i.e. HUFA PC species, and the PC/PE ratio [5]. Phosphatydilcholine and PE species were determined as described by HPLC-mass spectroscopy [33]. In short, 150 μL of the homogenized liver biopsy was centrifuged for 5 min at 7000 x g and 115 μL of the supernatant was incubated for 1 h at 37 °C with 0.4 mM 16:0-dimethyl PE (PEDM; Avanti Polar Lipids, Alabaster, AL, USA), 50 μM S-(5′-Adenosyl)-L-methionine (SAM; Sigma-Aldrich, St. Louis, MO, USA) and 0.34 μCi ^3^H-labeled SAM (Perkin Elmer, Waltham, MA, USA) in a final volume of 150 μL. After the incubation the lipids were extracted and separated with thin layer chromatography (TLC), and the PC spot was scraped and counted.

### Statistical analysis

As no previous studies in cats have been performed, the results of the study by DaCosta K.A et al. in humans were used to perform a power analysis to determine the minimal number of cats to be used in this study [9]. Based on the power calculations, the minimal number of female cats needed to demonstrate the effect of spaying on phosphatidylcholine-docosahexaenoic acid (PC-DHA) was six, based on the following data: mean PC—DHA before spay: 100 ± 25 nmol/ml; mean PC—DHA after spay: 59 ± 25 nmol/ml; alpha 0.05; power 0.88; paired t-test testing.

Data analysis was performed using IBM SPSS 22 statistic software (IBM Corporation Armonk, NY, United States of America).

The outcome variables plasma choline, PEMT, total liver PC content and PC:PE ratio were analysed using a linear mixed model with cat ID as random effect to take repeated observations within the subject into account. Variables time (before/after spaying/neutering) and gender (male/female) and the interaction between time and gender were used as explanatory variables. The outcome variables were log transformed to meet the model assumptions normality and the constance of variance. The akaike information criterion (AIC) was used to select the best model. Residuals plots were used to assess the validity of the model.

For outcome variables PC38:6 and PC40:6 the difference between both time moments (before/after spaying/neutering) and gender (male/female) were calculated as the model assumptions could not be met using the linear mixed effect model. A nonparametric Mann Whitney U test was applied on the differences to assess the difference in means between both genders.

The *P*-value <0.05 was used to assess statistical significance. Results are expressed as mean and standard deviation, if not indicated otherwise.

## Abbreviations

FHL: Feline hepatic lipidosis
HL: Hepatic lipidosis
CPD-choline: Cytidine diphosphate-choline
PEMT: Phosphatidylethanolamine N-methyltransferase
VLDL: Very low density lipoprotein
PC: Phosphatidylcholine
TAG: Triglycerides
DHA: Docosahexaenoic acid
HUFAs: Highly unsaturated acyl chains
PE: Phosphatidylethanolamine

## References

[1] Armstrong PJ, Blanchard G (2009). Hepatic lipidosis in cats. Vet Clin North Am Small Anim Pract.

[2] Center SA (2005). Feline hepatic lipidosis. Vet Clin North Am Small Anim Pract.

[3] Center SA, Crawford MA, Guida L, Erb HN, King J (1993). A retrospective study of 77 cats with severe hepatic lipidosis: 1975–1990. J Vet Intern Med.

[4] Pazak HE, Bartges JW, Cornelius LC, Scott MA, Gross K, Huber TL (1998). Characterization of serum lipoprotein profiles of healthy, adult cats and idiopathic feline hepatic lipidosis patients. J Nutr.

[5] DeLong CJ, Shen YJ, Thomas MJ, Cui Z (1999). Molecular distinction of phosphatidylcholine synthesis between the CDP-choline pathway and phosphatidylethanolamine methylation pathway. J Biol Chem.

[6] Vance DE, Vance DE, Vance JE (2002). Phospholipid biosynthesis in eukaryotes. Biochemistry of Lipids, Lipoproteins and Membranes.

[7] Vance DE, Ridgway ND (1988). The methylation of phosphatidylethanolamine. Prog Lipid Res.

[8] Li Z, Vance DE (2008). Phosphatidylcholine and choline homeostasis. J Lipid Res.

[9] da Costa KA, Sanders LM, Fischer LM, Zeisel SH (2011). Docosahexaenoic acid in plasma phosphatidylcholine may be a potential marker for in vivo phosphatidylethanolamine N-methyltransferase activity in humans. Am J Clin Nutr.

[10] Pani P, Porcu M, Columbano A, Dessi S, Ledda GM, Diaz G (1978). Differential effects of choline administration on liver microsomes of female and male rats. Exp Mol Pathol.

[11] Noga AA, Vance DE (2003). A gender-specific role for phosphatidylethanolamine N-methyltransferase-derived phosphatidylcholine in the regulation of plasma high density and very low density lipoproteins in mice. J Biol Chem.

[12] Zeisel SH (2000). Choline: an essential nutrient for humans. Nutrition.

[13] Da Silva AC, Guerios SMF, Monsao SR (1959). The domestic cat as a laboratory animal for experimental nutrition studies. VI. Choline deficiency. J Nutr.

[14] Anderson PA, Baker DH, Sherry PA, Corbin JE (1979). Choline-methionine interrelationship in feline nutrition. J Anim Sci.

[15] Schaeffer MC, Rogers QR, Morris JG (1982). Methionine requirement of the growing kitten, in the absence of dietary cystine. J Nutr.

[16] Verbrugghe A, Bakovic M (2013). Peculiarities of one-carbon metabolism in the strict carnivorous cat and the role in feline hepatic lipidosis. Nutrients.

[17] Li Z, Agellon LB, Vance DE (2005). Phosphatidylcholine homeostasis and liver failure. J Biol Chem.

[18] Vance JE, Vance DE (1985). The role of phosphatidylcholine biosynthesis in the secretion of lipoproteins from hepatocytes. Can J Biochem Cell Biol.

[19] Yao ZM, Vance DE (1988). The active synthesis of phosphatidylcholine is required for very low density lipoprotein secretion from rat hepatocytes. J Biol Chem.

[20] Yao ZM, Vance DE (1989). Head group specificity in the requirement of phosphatidylcholine biosynthesis for very low density lipoprotein secretion from cultured hepatocytes. J Biol Chem.

[21] Yao ZM, Vance DE (1990). Reduction in VLDL, but not HDL, in plasma of rats deficient in choline. Biochem Cell Biol.

[22] Buchman AL, Dubin MD, Moukarzel AA, Jenden DJ, Roch M, Rice KM, Gornbein J, Ament ME (1995). Choline deficiency: a cause of hepatic steatosis during parenteral nutrition that can be reversed with intravenous choline supplementation. Hepatology.

[23] Fischer LM, da Costa KA, Kwock L, Stewart PW, Lu TS, Stabler SP, Allen RH, Zeisel SH (2007). Sex and menopausal status influence human dietary requirements for the nutrient choline. Am J Clin Nutr.

[24] Resseguie M, Song J, Niculescu MD, da Costa KA, Randall TA, Zeisel SH (2007). Phosphatidylethanolamine N-methyltransferase (PEMT) gene expression is induced by estrogen in human and mouse primary hepatocytes. FASEB J.

[25] Young DL (1971). Estradiol- and testosterone-induced alterations in phosphatidylcholine and triglyceride synthesis in hepatic endoplasmic reticulum. J Lipid Res.

[26] National Research Council (NRC) (2006). Nutrient requirements of dogs and cats.

[27] Gardiner JE, Paton WD (1972). The control of the plasma choline concentration in the cat. J Physiol.

[28] Dieleman SJ, Bevers MM (1987). Effects of monoclonal antibody against PMSG administered shortly after the preovulatory LH surge on time and number of ovulations in PMSG/PG-treated cows. J Reprod Fertil.

[29] van Haaften B, Bevers MM, van den Brom WE, Okkens AC, van Sluijs FJ, Willemse AH, Dieleman SJ (1994). Increasing sensitivity of the pituitary to GnRH from early to late anoestrus in the beagle bitch. J Reprod Fertil.

[30] Buijtels JJ, Beijerink NJ, Kooistra HS, Dieleman SJ, Okkens AC (2006). Effects of gonadotrophin releasing hormone administration on the pituitary-ovarian axis in anoestrous vs ovariectomized bitches. Reprod Domest Anim.

[31] Ridgway ND, Vance DE (1992). Phosphatidylethanolamine N-methyltransferase from rat liver. Methods Enzymol.

[32] Testerink N, Ajat M, Houweling M, Brouwers JF, Pully VV, van Manen HJ, Otto C, Helms JB, Vaandrager AB (2012). Replacement of retinyl esters by polyunsaturated triacylglycerol species in lipid droplets of hepatic stellate cells during activation. PLoS One.

[33] Bleijerveld OB, Brouwers JF, Vaandrager AB, Helms JB, Houweling M (2007). The CDP-ethanolamine pathway and phosphatidylserine decarboxylation generate different phosphatidylethanolamine molecular species. J Biol Chem.

